# *Cryptosporidium baileyi* and *Mycoplasma synoviae* co-infection enhances *M. synoviae* colonization and aggravates tissue damage in chickens

**DOI:** 10.3389/fmicb.2026.1827782

**Published:** 2026-05-04

**Authors:** Yiqing Zhang, Yi Li, Zhanxing Wang, Qingfeng Zhou, Zhuanqiang Yan, Nanshan Qi, Shenquan Liao, Xuhui Lin, Minna Lv, Haiming Cai, Yongle Song, Xiangjie Chen, Yibin Zhu, Juan Li, Mingfei Sun

**Affiliations:** 1 State Key Laboratory of Swine and Poultry Breeding Industry, Guangdong Province Key Laboratory of Livestock Disease Prevention, Key Laboratory for Prevention and Control of Avian Influenza and Other Major Poultry Diseases, Ministry of Agriculture and Rural Affairs, Institute of Animal Health, Guangdong Academy of Agricultural Sciences, Guangzhou, Guangdong, China; 2 Wen’s Group Academy, Wen’s Foodstuffs Group Co., Ltd., Xinxing, Guangdong, China; 3 Joint Research Laboratory, Nansha, Guangdong, China

**Keywords:** co-infection, *Cryptosporidium baileyi*, damage, epidemiology, *Mycoplasma synoviae*

## Abstract

**Introduction:**

*Mycoplasma synoviae* (*M. synoviae*) is an economically significant pathogen that causes respiratory infections, synovitis, and arthritis in chickens, inflicting substantial economic losses on the global poultry industry. Its frequent co-infection with other respiratory pathogens, often exacerbates the resultant pathogenic damage. As an important respiratory pathogen, it remains unclear whether *Cryptosporidium baileyi* (*C. baileyi*) can cause co-infection with *M. synoviae* in commercial large-scale poultry farms and what synergistic pathogenic pattern exists between them.

**Methods:**

A total of 1,118 choanal cleft swab samples were collected from commercial chicken farms across eight regions in Guangdong province for the detection of *M. synoviae* and *C. baileyi*. The extracted DNA was analyzed by qPCR for *M. synoviae* and nested PCR for *C. baileyi*, respectively. Furthermore, a total of 90 one-day-old chicks confirmed free of *C. baileyi* and *M. synoviae* were randomly divided into six groups to establish a co-infection model and investigate the synergistic pathogenic effect pattern of the two pathogens.

**Results:**

The overall positive rates of *M. synoviae* and *C. baileyi* were 41.32% and 17.80%, respectively, with significant regional, city-level, and age-related variations. Samples from Western Guangdong and chickens over 45 days old showed the highest infection risks for both pathogens. The co-infection rate was 10.55%, and a significant positive association was observed between the two pathogens (OR = 2.44, *p* < 0.001). Co-infection risk increased markedly with age, especially in chickens older than 45 days. Chicken co-infection model was established to explore synergistic pathogenesis between *C. baileyi* and *M. synoviae*. Co-infection did not alter the prepatent period of *C. baileyi*, but significantly increased oocyst shedding peak and prolonged excretion time. Meanwhile, *C. baileyi* markedly elevated *M. synoviae* loads in the choanal cleft at multiple time points. Gross and histopathological examinations showed that co-infection aggravated laryngotracheal lesions caused by *C. baileyi*, and exacerbated footpad, joint and air sac lesions induced by *M. synoviae.* Overall, *C. baileyi* and *M. synoviae* exert mutual promoting effects on proliferation and pathogenicity in chickens.

**Discussion:**

The findings in the present study confirm the high prevalence of *M. synoviae*-*C. baileyi* co-infection in commercial poultry flocks and demonstrate that co-infection synergistically enhances the pathogenicity of both pathogens. These results fill an important knowledge gap in co-infection research, provide novel insights into the interaction mechanisms of multiple pathogens in poultry, and offer key scientific support for addressing complex disease challenges in modern poultry production.

## 1 Introduction

*Mycoplasma synoviae* was first reported as a major avian pathogen in 1954 (Feberwee et al., 2022). Following infection, it shows host pathological manifestations: in chickens, it mainly invades the respiratory system, clinically presenting with coughing, respiratory rales, and nasal discharge, while in turkeys, it specifically triggers infraorbital sinusitis (Bergeron et al., 2021). In addition to respiratory symptoms, *M. synoviae* is capable of inducing synovitis, airsacculitis, arthritis, and eggshell apex abnormalities (Han et al., 2024; Hu et al., 2025; Wang et al., 2025; Zare et al., 2025; Zhang et al., 2025). Infected flocks often suffer from reduced feed conversion efficiency, compromised carcass quality, declined egg production, and increased malformed egg rates (Landman and Feberwee, 2012; Kursa et al., 2019), which collectively result in considerable economic burdens for the global poultry industry.

*Mycoplasma synoviae* exhibits a global distribution with high prevalence in commercial poultry flocks, with transmission occurring primarily via vertical and horizontal pathways, and long-distance spread mediated by the trade of hatching eggs, day-old chicks, and juvenile birds (Ogino et al., 2011; Cizelj et al., 2015). A global epidemiological survey estimated that roughly one-third of poultry populations worldwide are infected with *M. synoviae*, with the highest prevalence rates reported in sub-Saharan Africa, and the pathogen is particularly prevalent in breeder flocks and layer hen populations (Chaidez-Ibarra et al., 2022). An epidemiological investigation targeting U. S. poultry, employing enzyme-linked immunosorbent assay (ELISA), detected a *M. synoviae* prevalence rate of 75.6% (Derksen et al., 2018). Similarly, *M. synoviae* is endemic in China: a cross-provincial surveillance study conducted between August 2020 and June 2021 identified 324 positive samples out of 487 field isolates, corresponding to an overall positivity rate of 66.53% (Wei et al., 2023). The persistently high prevalence of *M. synoviae* highlights its substantial threat to the sustainability of the global poultry industry. To date, no therapeutic agents have been shown to completely eliminate *M. synoviae* infections in affected flocks. Thus, a comprehensive understanding of the epidemiology and pathogenic mechanisms underlying *M. synoviae* infection is essential for the development of effective control and prevention strategies.

*Mycoplasma synoviae* typically establishes chronic or subclinical infections in poultry under clinical settings. However, its pathogenic potential is markedly exacerbated upon co-infection with other bacterial or viral pathogens. Clinical diagnostic investigations frequently detect *M. synoviae* in polymicrobial infections involving a diverse array of bacteria and viruses (Kreizinger et al., 2018). Between 2017 and 2019, seven episodes of mild-to-severe respiratory disease outbreaks were documented on commercial poultry farms in Slovenia. Laboratory analyses of clinical samples from all these outbreaks confirmed concurrent infections with infectious laryngotracheitis virus (ILTV) and *M. synoviae* (Zorman et al., 2021). Furthermore, experimental co-infection studies involving *M. synoviae* and infectious bronchitis virus (IBV) have demonstrated that IBV can potentiate the pathogenicity of *M. synoviae*. Transcriptomic sequencing analyses revealed that co-infection with IBV and *M. synoviae* elicits more pronounced immune dysregulation, excessive inflammatory responses, and severe cytotoxic damage in infected birds (Kamathewatta et al., 2024). *C. baileyi* is a globally prevalent, economically significant pathogen that severely impairs poultry health, primarily inducing pathological lesions in the respiratory tract, intestinal tract, and bursa of Fabricius (Yuan et al., 2014; Wu et al., 2021). As a cause of chronic wasting disease, *C. baileyi* exerts immunosuppressive effects on its avian host, thereby predisposing them to secondary or mixed infections with respiratory, enteric, and bursal pathogens. For instance, broilers co-infected with *C. baileyi* and chicken anemia virus (CAV) exhibit significant growth retardation and elevated mortality rates (Dobos-Kovacs et al., 1994). Notably, in one-day-old chicks vaccinated against H5N1 avian influenza virus (AIV), *C. baileyi* infection induces bursal atrophy and consequent immunosuppression, which in turn enhances the susceptibility of these chicks to AIV infection (Hao et al., 2008). Both *M. synoviae* and *C. baileyi* are clinically and economically important respiratory pathogens in poultry production systems. Nevertheless, clinical reports documenting their co-occurrence and synergistic interactions in field conditions remain scarce. Therefore, the potential synergistic pathogenicity between *M. synoviae* and *C. baileyi*, as well as the underlying molecular and immunological mechanisms, merits in-depth investigation.

In the present study, choanal cleft samples were collected from commercial-scale poultry farms distributed across multiple regions of Guangdong province. A panel of molecular biological methods was employed to systematically determine the prevalence and co-infection profiles of *M. synoviae* and *C. baileyi*. Epidemiological analyses revealed a notable high co-infection rate of the two pathogens, along with a statistically significant positive correlation between their individual prevalence and co-infection occurrence. To further dissect the interactive roles of these pathogens during synergistic pathogenic progression, a specific animal infection model of *M. synoviae*-*C. baileyi* co-infection was developed. Experimental results demonstrated that *M. synoviae* and *C. baileyi* could synergistically exacerbate host tissue lesions and pathological damage in infected birds. Collectively, these findings advance the current understanding of the epidemiological features and synergistic mechanisms underlying co-infections, thereby laying a scientific foundation for the formulation of targeted prevention and control strategies in the poultry industry.

## 2 Materials and methods

### 2.1 Ethical approval

The animal experimental procedure is in strict accordance with the recommendations of the Ethical Review Committee (No. PT-2024033) of the Institute of Animal Health, Guangdong Academy of Agricultural Sciences, China. The samples were collected with the prior consent of the farm owner.

### 2.2 Strains

*Cryptosporidium baileyi*: Preserved by the Laboratory of Parasitology, Institute of Animal Health, Guangdong Academy of Agricultural Sciences. *M. synoviae* attenuated strain (MSH): Purchased from the Australian Biological Resources Company (1,000 doses/vial; Lot No.: MSH190121A). *M. synoviae* virulent strain (MSW): Provided by Wen’s Group Academy, Wen’s Foodstuffs Group Co., Ltd. The titer and activity were measured before use to ensure effective infection.

### 2.3 Sample collection

**Figure 1:**
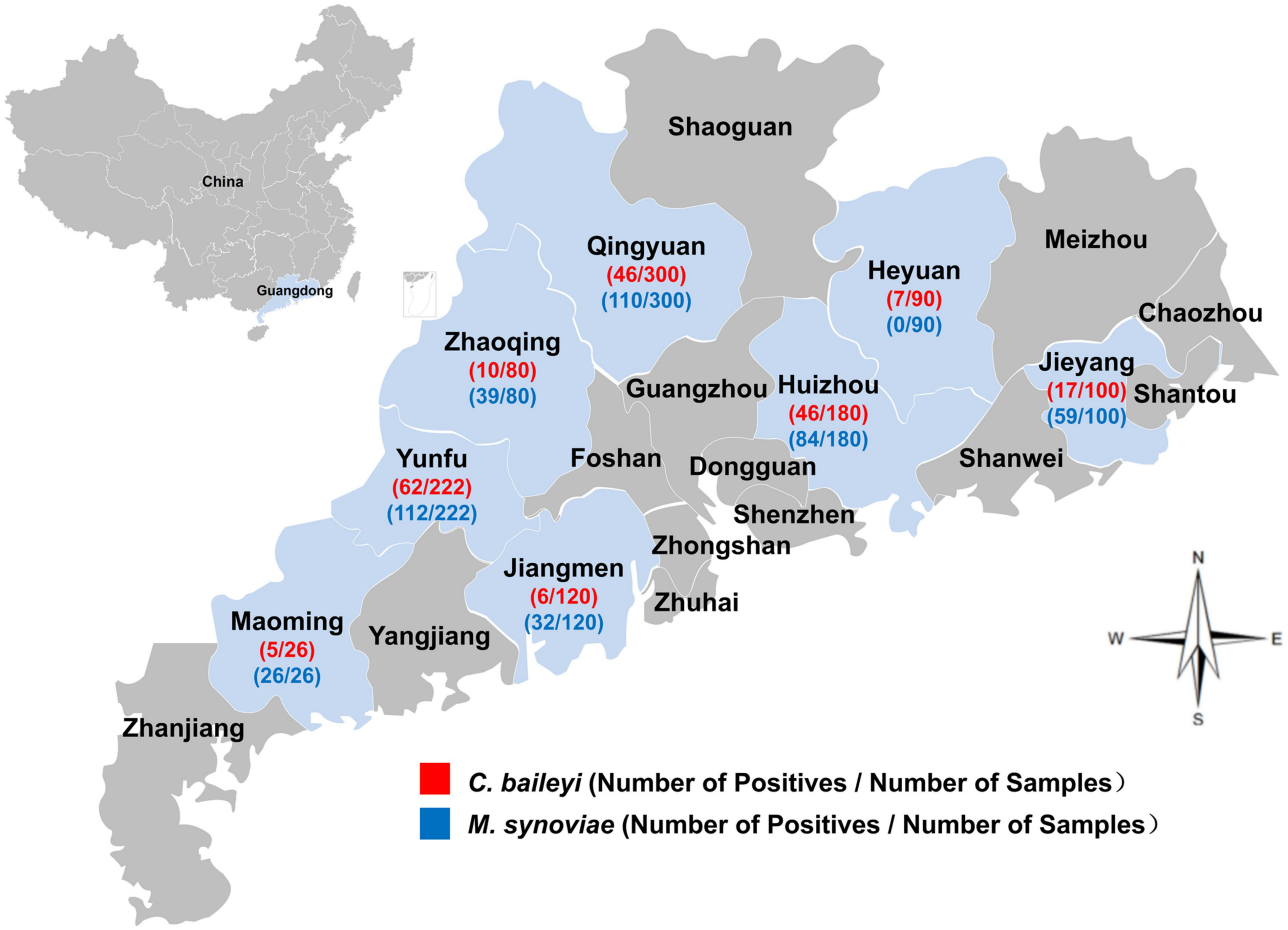
Occurrence of *M. synoviae* and *C. baileyi* in choanal cleft swab samples from chickens in distinct regions in Guangdong province, China.

A total of 1,118 choanal cleft swab samples were collected from commercial chicken farms across eight regions in Guangdong province, covering the eastern, western, northern, and Pearl River Delta regions (Figure 1). Three to five sampling sites were selected per region. At each sampling site, five chickens per poultry house were sampled using the five-point sampling method. Choanal cleft swabs were collected by gently swabbing the palatine cleft and supraglottic area 5–8 times using sterile cotton swabs. The swabs were subsequently transferred into 1.5 mL centrifuge tubes prefilled with 1 mL of sterile phosphate-buffered saline (PBS) and subjected to immediate labeling. All harvested samples were maintained at −20 °C pending nucleic acid extraction. A full set of metadata, encompassing sampling date, geographic origin, chicken breed, age (days) and rearing regimen were captured in standardized data acquisition forms.

### 2.4 Methods for detection of *M. synoviae* and *C. baileyi*

DNA was extracted using the E. Z. N. A.^®^ Stool DNA Kit (Omega, D4015), and the obtained DNA samples were stored at −20 °C until further analysis. *C. baileyi* was detected by a nested PCR method targeting its small subunit rRNA (*SSU* rRNA) gene (Ryan et al., 2003). The first-round PCR amplified a 763 bp fragment with the following primers: Forward: 5′-GACATATCATTCAAGTTTCTGACC-3′, Reverse: 5′-CTGAAGGAGTAAGGAACAACC-3′. The second-round PCR yielded a 587 bp fragment using the following primers: Forward: 5′-CCTATCAGCTTTAGACGGTAGG-3′, Reverse: 5′-TCTAAGAA TTTCACCTCTGACTG-3′. Each PCR reaction contained 12.5 μL of 2 × rTaq Mix (TAKARA) and primers at a final concentration of 5 μM each. The PCR condition was as follows: an initial denaturation at 94 °C for 5 min; 35 cycles of denaturation at 94 °C for 30 s, annealing at 58 °C for 30 s, and extension at 72 °C for 1 min; followed by a final extension at 72 °C for 10 min.

*Mycoplasma synoviae* was detected by qPCR targeting the spo0B-associated GTP binding protein (*obg*) gene, using primers, probes, and reaction conditions as described previously (Chen et al., 2025). The primers set was able to detect both MSH and MSW strains: Forward primer MS-uni-F312: 5′-TGCCGATGTTATTAAAGAAAATCATC-3′, Reverse primer MS-uni-R432: 5′-CATTCCATTTTCGGCAATT CTAG-3′. The probe for the MSH strain, MS367-PV (5′-TTTCCT CTTCTGCCTTT-3′), was labeled with VIC at the 5′ end and an MGB quencher at the 3′ end. The probe for the MSW strain, MS367-PW (5′-ATTTCCTCTTCCGCCTT-3′), was labeled with 6-FAM at the 5′ end and an MGB quencher at the 3′ end. Each qPCR reaction was performed in a 20 μL volume containing 10 μL of 2 × Probe qPCR Mix (TAKARA), 1.8 μL of MS-uni-F312, 1.8 μL of MS-uni-R432, 0.4 μL of MS367-PW, 0.2 μL of MS367-PV, 2 μL of DNA template, and nuclease-free ddH₂O to adjust the final volume. The reaction condition was as follows: 95 °C for 30 s; followed by 45 cycles of 95 °C for 10 s and 60 °C for 30 s.

### 2.5 Animal experiment on the synergistic pathogenicity of *M. synoviae* and *C. baileyi*

A total of 90 one-day-old Mahuang broilers chicks, confirmed to be free of *C. baileyi* and *M. synoviae* infections, were weighed and randomly divided into six groups with 15 chicks per group. The groups were designated as follows: Group 1 (control, Ctrl), Group 2 (*C. baileyi* single infection, *C. baileyi*), Group 3 (MSH single infection, MSH), Group 4 (MSW single infection, MSW), Group 5 (Co-infection with *C. baileyi* and MSH, *C. baileyi* + MSH), Group 6 (Co-infection with *C. baileyi* and MSW, *C. baileyi* + MSW). At 3 days of age, chicks in Groups 2, 5, and 6 were orally inoculated with 1.5 × 10^5^ *C. baileyi* oocysts, whereas the remaining groups received an equal volume of sterile PBS. At 4 days of age, chicks in Groups 3 and 5 were intranasally administered 0.3 mL of an MSH bacterial suspension (concentration: 1.0 × 10^6.5^ CCU/mL), corresponding to an inoculation dose of 3.0 × 10^5.5^ CCU per chick. Chicks in Groups 4 and 6 were intranasally administered 0.1 mL of an MSW bacterial suspension (concentration: 1.0 × 10^9^ CCU/mL), resulting in an inoculation dose of 1.0 × 10^8^ CCU per chick. The remaining groups were intranasally given an equivalent volume of sterile saline. Throughout the entire experiment, chickens in each group were reared separately in thoroughly disinfected and isolated facilities with physical separation maintained between groups. The environment was sprayed with disinfectant at fixed times daily, and the birds had free access to feed and water.

From the day of *C. baileyi* oocyst inoculation onward, fecal samples were collected daily from each group. Oocysts were concentrated using the saturated sucrose solution flotation method and quantified using a hemocytometer (Bukhari and Smith, 1995; Lichtmannsperger et al., 2019). At 7, 14, and 21 days post-infection (dpi) with *M. synoviae*, choanal cleft swabs were collected from all chicks, and the colonization levels of MSH and MSW were quantified by qPCR. At 14 dpi with *M. synoviae*, three chicks were randomly selected from each group and humanely euthanized. Laryngeal and tracheal tissues were harvested, fixed in 10% neutral buffered formalin, embedded in paraffin, sectioned at 4–5 μm, and stained with hematoxylin and eosin (H&E) according to standard protocols (Withoeft et al., 2025). Histopathological lesions were observed and evaluated under a light microscope (Nikon Eclipse Ci-L, Japan). At 21 dpi, all remaining chicks were humanely euthanized, and systematic lesion scoring was performed on the air sacs, foot pads, and joints (Lockaby et al., 1998; Moronato et al., 2018).

### 2.6 Statistical analysis

Statistical analysis was performed using SPSS 23.0 software, and intergroup differences were assessed by the *χ*^2^ test. A *p*-value greater than 0.05 was considered statistically non-significant. If the *p*-value ranged between 0.01 and 0.05, the difference was deemed statistically significant. When the *p*-value was less than 0.01, the difference was considered highly statistically significant.

## 3 Results

### 3.1 Co-infection of *M. synoviae* and *C. baileyi* is prevalent in intensive poultry farms across Guangdong province

A total of 1,118 choanal cleft swab samples were collected from intensive chicken farms across different regions of Guangdong province to detect *C. baileyi* and *M. synoviae*. The overall infection status of the two pathogens differed significantly (Table 1). The overall positive rate for *C. baileyi* infection was 17.80% (199/1,118, 95% CI: 15.60–20.00%). In contrast, the infection rate of *M. synoviae* was markedly higher, with an overall positive rate of 41.32% (462/1118, 95% CI: 38.40–44.20%). Analysis of regional infection data showed significant geographical variations in the positive rate of *C. baileyi*. The highest positive rate was detected in Western Guangdong (27.02%, 67/248), which was significantly higher than those in Eastern Guangdong (12.63%, 24/190), Northern Guangdong (15.33%, 46/300), and the Pearl River Delta region (16.32%, 62/380) (all *p* < 0.05). Using Eastern Guangdong as the reference group, the odds ratio (OR) for *C. baileyi* infection in Western Guangdong was 2.56 (*p* < 0.001), suggesting a significantly elevated infection risk. Significant geographical differences were also observed in the distribution of *M. synoviae* infection. The highest positive rate was found in Western Guangdong (55.65%, 138/248), followed by the Pearl River Delta (40.79%, 155/380), Northern Guangdong (36.67%, 110/300), and Eastern Guangdong (31.05%, 59/190). The positive rate in Western Guangdong was significantly higher than in the other three regions (all *p* < 0.05). For infection risk, with Eastern Guangdong as the reference group, the OR was 2.78 for Western Guangdong (*p* < 0.001) and 1.53 for the Pearl River Delta (*p* = 0.020), both indicating a significantly increased risk of infection.

**Table 1:** Prevalence of *M. synoviae* and *C. baileyi* in different regions of Guangdong province.

Factor	Category	Sample Size	No. *C. baileyi* Positive (Positivity Rate %, 95 CI)	OR (95% CI)	*p*-value	No. *M. synoviae* Positive (Positivity Rate %, 95 CI)	OR (95% CI)	*p*-value	No. Co-infection (Positivity Rate %, 95 CI)	OR (95% CI)	*p*-value
Region	Eastern GD	190	24 (12.63%, 7.80–17.40)	1	–	59 (31.05%, 24.50–37.70)	1	–	9 (4.74%, 2.20–8.78)	1	–
	Western GD	248	67 (27.02%, 21.40–32.60)	2.56 (1.53–4.28)	<0.001	138 (55.65%, 49.40–61.80)	2.78 (1.91–4.06)	<0.001	44 (17.74%, 13.16–23.19)	4.32 (2.04–9.15)	<0.001
	Northern GD	300	46 (15.33%, 11.30–19.30)	1.25 (0.74–2.12)	0.402	110 (36.67%, 31.20–42.20)	1.29 (0.88–1.88)	0.192	24 (8.00%, 5.21–11.68)	1.75 (0.79–3.86)	0.165
	Pearl River Delta	380	62 (16.32%, 12.60–20.00)	1.35 (0.81–2.23)	0.247	155 (40.79%, 35.80–45.80)	1.53 (1.07–2.19)	0.020	41 (10.79%, 7.88–14.44)	2.43 (1.15–5.12)	0.020
Location	Jieyang	100	17 (17.00%, 9.60–24.40)	1	–	59 (59.00%, 49.30–68.70)	1	–	9 (9.00%, 4.18–16.39)	1	–
	Heyuan	90	7 (7.78%, 2.20–13.40)	0.41 (0.16–1.05)	0.064	0 (0.00%, 0–4.00)	–	<0.001	0 (0.00%, 0–4.03)	–	<0.001
	Yunfu	222	62 (27.93%, 22.00–33.80)	1.89 (1.03–3.47)	0.039	112 (50.45%, 43.80–57.20)	0.71 (0.43–1.16)	0.169	42 (18.92%, 13.98–24.74)	2.36 (1.09–5.09)	0.029
	Maoming	26	5 (19.23%, 4.00–34.40)	1.16 (0.38–3.52)	0.792	26 (100.00%, 86.80–100)	–	<0.001	2 (7.69%, 0.95–25.13)	0.84 (0.17–4.17)	0.835
	Qingyuan	300	46 (15.33%, 11.30–19.30)	0.88 (0.48–1.63)	0.690	110 (36.67%, 31.20–42.20)	0.40 (0.25–0.65)	<0.001	24 (8.00%, 5.21–11.68)	0.88 (0.39–1.96)	0.753
	Jiangmen	120	6 (5.00%, 1.10–8.90)	0.26 (0.09–0.69)	0.007	32 (26.67%, 18.70–34.70)	0.25 (0.14–0.45)	<0.001	2 (1.67%, 0.20–5.91)	0.17 (0.04–0.81)	0.026
	Zhaoqing	80	10 (12.50%, 5.30–19.70)	0.70 (0.30–1.63)	0.405	39 (48.75%, 37.70–59.90)	0.66 (0.36–1.20)	0.173	6 (7.50%, 2.80–15.61)	0.82 (0.28–2.41)	0.718
	Huizhou	180	46 (25.56%, 19.30–31.90)	1.68 (0.90–3.12)	0.103	84 (46.67%, 39.40–54.00)	0.61 (0.37–1.00)	0.050	33 (18.33%, 13.02–24.74)	2.27 (1.04–4.96)	0.040
Age (days)	≤25 d	240	26 (10.83%, 6.90–14.70)	1	–	5 (2.08%, 0.30–3.90)	1	–	2 (0.83%, 0.10–2.98)	1	–
	25 ~ 45 d	295	46 (15.59%, 11.50–19.70)	1.52 (0.91–2.54)	0.109	37 (12.54%, 8.70–16.30)	6.75 (2.62–17.41)	<0.001	3 (1.02%, 0.21–2.94)	1.22 (0.20–7.37)	0.827
	>45 d	583	127 (21.78%, 18.50–25.10)	2.29 (1.46–3.60)	<0.001	420 (72.04%, 68.40–75.60)	121.29 (48.90–300.80)	<0.001	113 (19.38%, 16.23–22.87)	28.84 (7.09–117.37)	<0.001
Total		1,118	199 (17.80%, 15.60–20.00)	–	–	462 (41.32%, 38.40–44.20)	–	–	118 (10.55%, 8.78–12.55)	2.44 (1.83–3.25)	<0.001

Analysis of infection data across different cities demonstrated significant variations in the positive rate of *C. baileyi*. Among the eight cities surveyed, the positive rate for *C. baileyi* ranged from 5.00 to 27.93%. Yunfu had the highest positive rate (27.93%, 62/222), followed by Huizhou (25.56%, 46/180) and Maoming (19.23%, 5/26). Jiangmen recorded the lowest positive rate at 5.00% (6/120). The positive rate of *M. synoviae* also varied markedly among cities, ranging from 0 to 100%. Maoming had the highest positive rate (100%, 26/26), followed by Jieyang (59.00%, 59/100) and Yunfu (50.45%, 112/222), whereas no *M. synoviae* positive samples were detected in Heyuan. Using Jieyang as the reference group, the infection risk was significantly lower in Qingyuan (OR = 0.40, *p* < 0.001) and Jiangmen (OR = 0.25, *p* < 0.001). OR values could not be calculated for Heyuan (no positive cases) or Maoming (100% positive rate), but significant differences were confirmed by statistical tests (both *p* < 0.001).

Analysis of infection patterns across different age groups revealed that the positivity rate for *C. baileyi* gradually increased with chicken age. The positivity rates were 10.83% (26/240), 15.59% (46/295), and 21.78% (127/583) in chickens aged ≤25 days, 25–45 days, and >45 days, respectively. The rate in the >45-day group was significantly higher than that in the ≤25-day group (*p* < 0.001). Using the ≤25-day group as the reference, the odds ratio (OR) for the >45-day group was 2.29 (*p* < 0.001), indicating an elevated infection risk. For *M. synoviae*, highly significant differences in positivity rates were observed among age groups: 2.08% (5/240) in the ≤25 day group, 12.54% (37/295) in the 25–45 day group, and 72.04% (420/583) in the >45 day group. Statistically significant differences were observed between all pairwise age groups (*p* < 0.001). Relative to the ≤25 day reference group, the OR was 6.75 for the 25-45-day group and 121.29 for the >45-day group (both *p* < 0.001), demonstrating that the risk of *M. synoviae* infection increased markedly with age, especially in chickens older than 45 days.

The co-infection profiles of *C. baileyi* and *M. synoviae* are summarized in Table 1. Among the 1,118 chicks subjected to pathogen detection, 118 were confirmed to be co-infected with both pathogens, corresponding to an overall co-infection rate of 10.55% (118/1,118). Statistical analysis yielded an OR of 2.44 (95% confidence interval [95% CI]: 1.83–3.25, *p* < 0.001), indicating that chickens infected with *C. baileyi* were 2.44 times more likely to be concurrently infected with *M. synoviae* than *C. baileyi*-negative individuals. Collectively, these results demonstrate a statistically significant association between infection with the two pathogens.

Furthermore, age-stratified analysis indicated that the co-infection rate in chickens older than 45 days was 19.38%, with an OR of 28.84 (95% CI: 7.09–117.37, *p* < 0.001) compared with those aged ≤25 days. This finding underscores a strong positive correlation between host age and susceptibility to *C. baileyi*–*M. synoviae* co-infection.

### 3.2 *M. synoviae* enhances *C. baileyi* proliferation in chickens and prolongs oocyst shedding

**Figure 2:**
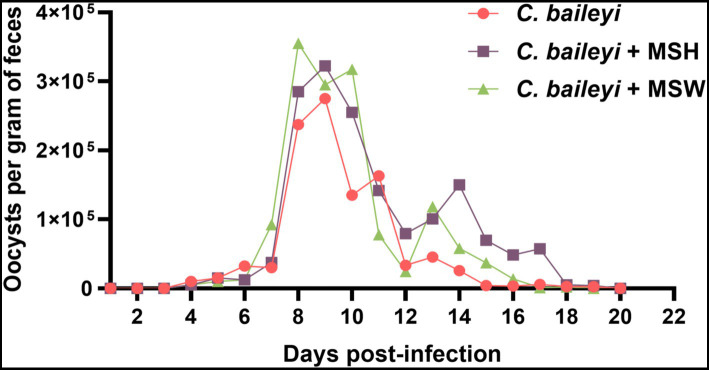
Effect of *M. synoviae* strains with varying virulence on oocyst shedding in *C. baileyi* infected chickens.

Given the epidemiological association observed between *M. synoviae* and *C. baileyi*, we established a co-infection animal model to investigate the synergistic pathogenic effects of these two pathogens in chickens. Subsequent to *C. baileyi* infection in chicks, fresh fecal samples were collected daily and subjected to oocysts examination using the saturated sucrose flotation technique. Oocysts were detected in fecal samples from all three experimental groups (*C. baileyi*, *C. baileyi* + MSH, and *C. baileyi* + MSW) from 3 dpi, indicating that *M. synoviae* co-infection does not significantly affect the prepatent period of *C. baileyi* (Figure 2). Quantitative analysis of fecal oocyst shedding demonstrated that the peak of oocyst excretion in all three groups occurred between 10 dpi and 11 dpi. During this peak phase, the fecal oocyst load in *C. baileyi* + MSH and *C. baileyi* + MSW groups was significantly higher than that in *C. baileyi* single infection group. Notably, the peak oocyst shedding in the *C. baileyi* + MSW group occurred earlier than that in the *C. baileyi* single infection group and the *C. baileyi* + MSH group. Furthermore, after the peak of oocyst shedding, the oocyst counts in *C. baileyi* single infection group declined rapidly, becoming nearly undetectable by 15 dpi. In contrast, the *C. baileyi* + MSH and *C. baileyi* + MSW groups maintained significantly higher oocyst counts than the *C. baileyi* single infection group after the peak period. Specifically, oocyst shedding in the *C. baileyi* + MSW group persisted until 17 dpi, while in the *C. baileyi* + MSH group, it continued until 18 dpi. These results indicate that co-infection with *M. synoviae* (regardless of strain) enhances the *in vivo* proliferation of *C. baileyi* and prolongs the duration of oocyst shedding during *C. baileyi* infection.

### 3.3 *M. synoviae* aggravates laryngotracheal lesions caused by *C. baileyi* infection in chickens

**Figure 3:**
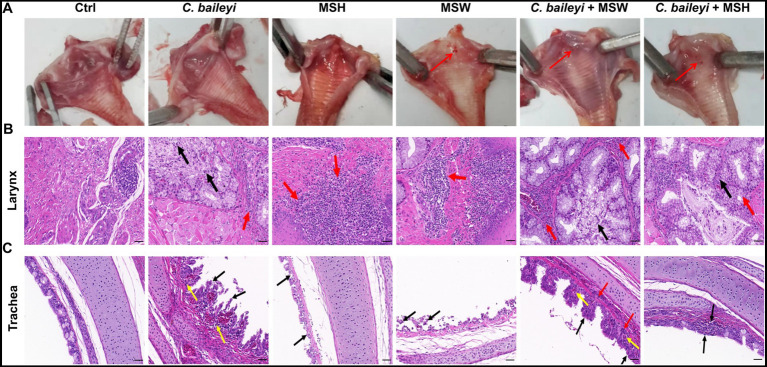
Pathological effects of *M. synoviae* on the larynx and trachea during co-infection with *C. baileyi*. **(A)** Gross lesions in the larynx and trachea. **(B)** Histopathological damage to the larynx (H&E staining). **(C)** Histopathological damage to the trachea (H&E staining).

To evaluate the pathological effects of co-infection on the larynx and trachea, all groups were subjected to necropsy and histopathological examination at 14 dpi. Gross lesion observation showed no obvious inflammation or hemorrhage in the laryngeal tissues of the control group. Mild inflammation was detected only in the group infected with *C. baileyi* alone. Infection with *M. synoviae* alone (either strain MSH or MSW) did not cause obvious laryngeal lesions. However, scattered hemorrhagic foci were observed in the group co-infected with *C. baileyi* and MSH. Furthermore, the group co-infected with *C. baileyi* and MSW showed marked inflammatory responses in the anterior larynx, with hemorrhagic lesions more extensive than those in the group infected with MSW alone (Figure 3A).

Histopathological examination further corroborated these observations. The laryngeal structure in the control group remained intact, with no pathological changes. Birds infected with *C. baileyi* alone exhibited mild glandular epithelial exfoliation and sparse inflammatory cell infiltration. Similarly, mild inflammatory cell infiltration was observed in groups infected with either MSH or MSW alone. In contrast, both co-infected groups (*C. baileyi* + MSH and *C. baileyi* + MSW) showed marked glandular epithelial exfoliation accompanied by focal inflammatory cell infiltration in the glandular interstitium (Figure 3B). Tracheal tissues displayed varying degrees of pathological damage across all infected groups. Chicken infected with *C. baileyi* alone exhibited extensive mucosal epithelial injury, characterized by focal epithelial exfoliation and mild inflammatory infiltration. In the group co-infected with MSH, the mucosal epithelium was attenuated, with concurrent focal inflammatory infiltration. In the group co-infected with MSW, epithelial cell arrangement was disorganized, accompanied by mild vascular congestion and dilation (Figure 3C). No abnormal lesions were observed in the tracheal tissues of the control group. Collectively, these histopathological findings demonstrate that co-infection with *M. synoviae* and *C. baileyi* significantly exacerbates laryngeal and tracheal lesions in chickens compared with *C. baileyi* single infection.

### 3.4 *C. baileyi* enhances the pathogen load of *M. synoviae* following dual infection

**Figure 4:**
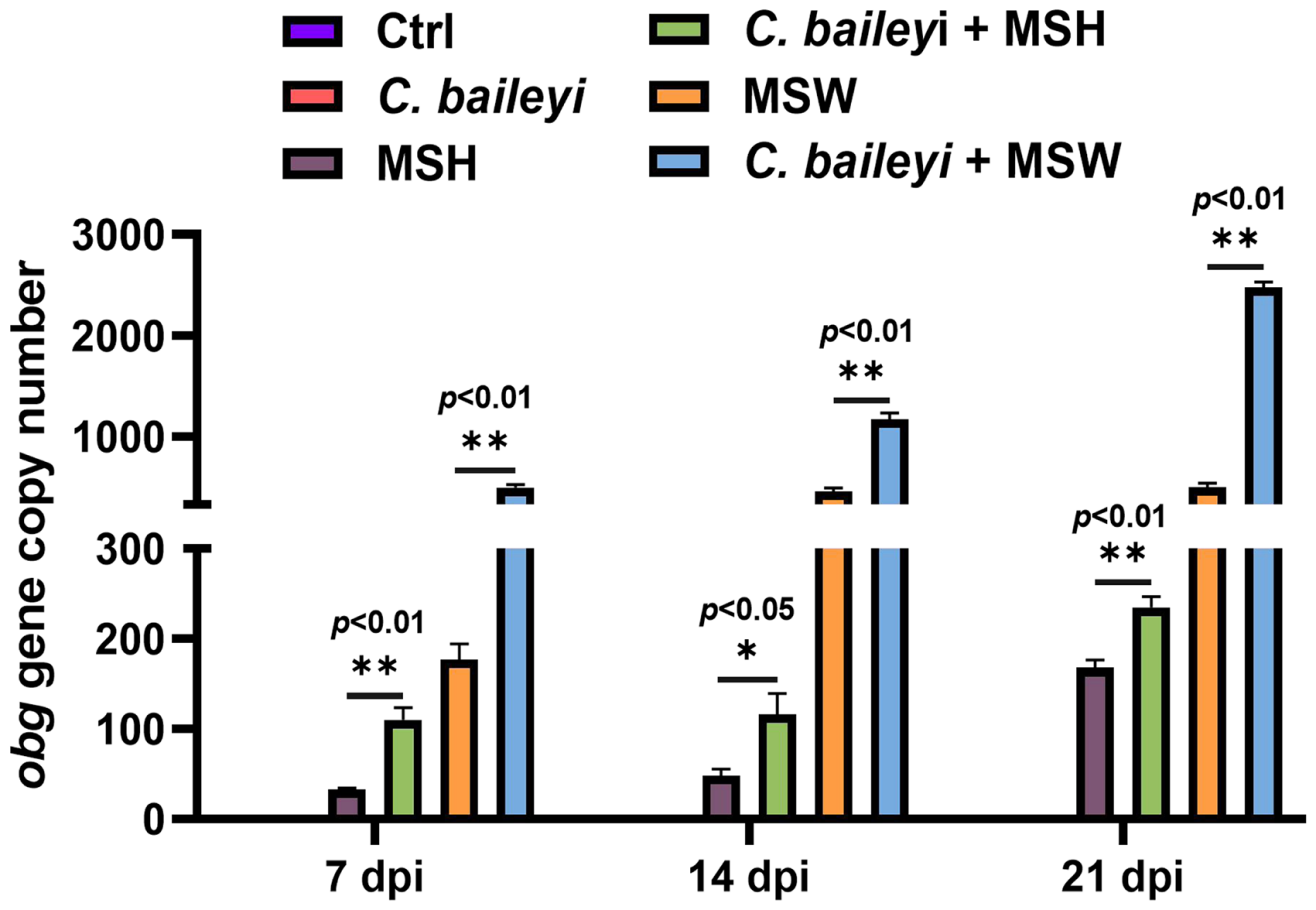
Colonization of *M. synoviae* in chickens during co-infection with *C. baileyi*.

To evaluate the effect of *C. baileyi* infection on the colonization load of *M. synoviae*, swabs were collected from the posterior choanal cleft of chickens in each group at 7, 14, and 21 dpi with *M. synoviae*, and the pathogen load was quantified by qPCR. qPCR analysis showed that *M. synoviae* was not detected at any time point in either the control group or the *C. baileyi* single-infection group. In groups inoculated with *M. synoviae*, the pathogen load increased gradually over time (Figure 4). Notably, the *M. synoviae* load in the co-infection groups was significantly higher than that in the corresponding single-infection groups at all time points. Specifically, the MSH load in the *C. baileyi* + MSH co-infection group was significantly higher than that in the MSH single-infection group. Similarly, the MSW load in the *C. baileyi* + MSW co-infection group was significantly higher than that in the MSW single-infection group (Figure 4). These findings demonstrate that co-infection with *C. baileyi* significantly increases the colonization load of *M. synoviae* in chickens, regardless of whether the *M. synoviae* strain is virulent or attenuated.

### 3.5 *C. baileyi* exacerbates lesions induced by *M. synoviae* in chicken foot pads and air sacs

**Figure 5:**
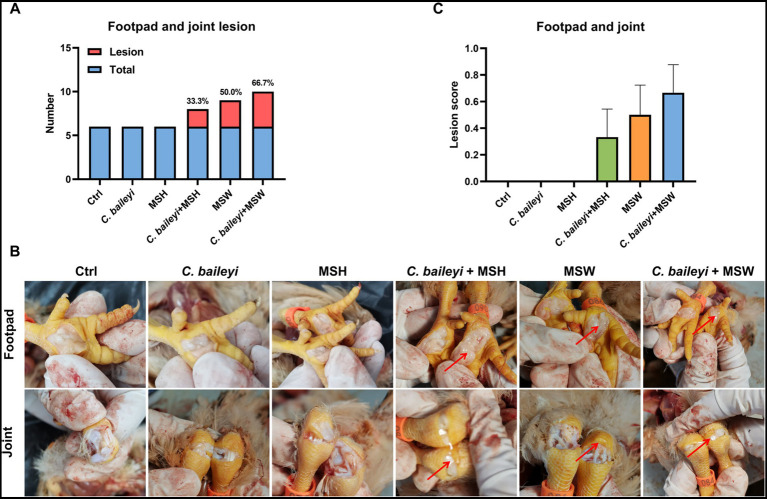
Pathological effects of *M. synoviae* on the footpad and joint during co-infection with *C. baileyi*. **(A)** Number of chickens exhibiting footpad lesions in different experimental groups. **(B)** Lesions in the footpad and joints induced by *M. synoviae* during co-infection with *C. baileyi*. **(C)** Lesion scores for footpad damage in different experimental groups.

At 21 dpi with *M. synoviae*, lesions in the footpads, joints, and air sacs of chickens in each group were evaluated. No obvious pathological changes in the footpads or joints were observed in the control group, the *C. baileyi* single-infection group, or the MSH single-infection group. In the *C. baileyi* + MSH co-infection group, 33.30% (2/6) of chickens showed mild footpad swelling (Figures 5A,B). In the MSW single-infection group, 50% (3/6) of chickens displayed a small amount of pale yellow purulent exudate in the footpads accompanied by joint swelling. In contrast, lesions in the *C. baileyi* + MSW co-infection group were more severe, with 66.70% (4/6) of chickens exhibiting marked footpad edema. Upon necropsy, large volumes of pale yellow fluid were observed, along with joint effusion and occasional hemorrhage (Figure 5B). Lesion scoring confirmed no pathological alterations in the control, *C. baileyi*, and MSH groups. The most severe lesions were found in the *C. baileyi* + MSW group, followed by the MSW group and the *C. baileyi* + MSH group (Figure 5C).

**Figure 6:**
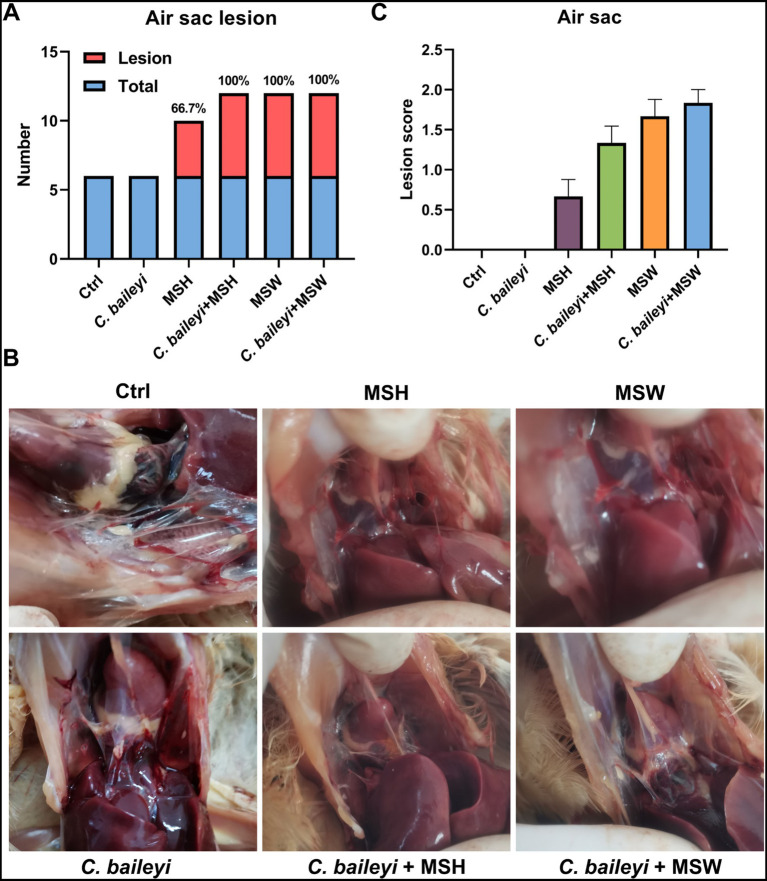
Pathological effects of *M. synoviae* on the air sac during co-infection with *C. baileyi*. **(A)** Number of chickens exhibiting air sac lesions in different experimental groups. **(B)** Air sac lesions induced by *M. synoviae* during co-infection with *C. baileyi*. **(C)** Lesion scores for air sac damage in different experimental groups.

Air sac lesions of varying severity were observed in all groups except the control and the *C. baileyi* single-infection groups. In the MSH single-infection group, 66.70% (4/6) of chickens showed decreased air sac transparency with a small amount of caseous exudate. In the MSW single-infection group, all chickens (100%, 6/6) displayed turbid air sacs accompanied by substantial caseous exudate accumulation and extensive focal caseous deposits. Notably, all chickens in both co-infection groups (*C. baileyi* + MSH and *C. baileyi* + MSW) exhibited more severe air sac lesions (100%, 6/6). Among these, the *C. baileyi* + MSW group exhibited the most severe pathological changes, characterized by marked air sac thickening and extensive caseous exudation (Figures 6A,B). Lesion scoring confirmed no obvious pathological alterations in the control and *C. baileyi* groups. The most severe lesions were detected in the *C. baileyi* + MSW group, followed by the MSW group, and lesions in the *C. baileyi* + MSH group were more severe than those in the MSH group (Figure 6C). Collectively, these results demonstrate that co-infection with *C. baileyi* and *M. synoviae* significantly exacerbates footpad, joint, and air sac lesions induced by *M. synoviae* single infection.

## 4 Discussion

*Mycoplasma synoviae* and *C. baileyi* are important pathogens associated with respiratory diseases in poultry, and infections often occur concurrently with other pathogens. To date, however, studies focusing on the co-infection and synergistic pathogenicity of *M. synoviae* and *C. baileyi* remain limited. This study provides a comprehensive overview of the epidemiological patterns of *C. baileyi* and *M. synoviae* infections in Guangdong province, a major poultry-producing region in China, and further explores the pathological alterations induced by their co-infection. The findings presented here will enhance our understanding of the epidemiological linkage and synergistic pathogenic effects between *M. synoviae* and *C. baileyi* under clinical production conditions, providing a novel perspective for interpreting complex multi-pathogen infection syndromes in poultry. These results also present new challenges to current prevention and control strategies, especially with regard to the evaluation of vaccine efficacy.

Infections with respiratory pathogens represent a major threat to poultry health, impeding the sustainable development of the poultry industry and causing substantial economic losses (Yehia et al., 2023). *M. synoviae* is a key pathogen causing avian respiratory diseases and is widely distributed in poultry farms worldwide (Zhao et al., 2025; Wei et al., 2025). For example, the seropositivity rate of *M. synoviae* in Canadian laying flocks has been reported to reach 57.90%, while a previous study across 16 provinces in China detected a positivity rate of 96.31% in chicken synovial fluid samples, with flock level positivity ranging from 10% to 100% (Sun et al., 2017; Bergeron et al., 2021). The present survey revealed an overall *M. synoviae* positivity rate of 41.32% in Guangdong province, which is consistent with the global epidemiological profile of this pathogen and highlights its endemic status in the local poultry industry. Notably, our data confirmed a highly significant positive correlation between *M. synoviae* infection rate and poultry age (the infection rate was only 2.08% in birds aged ≤25 days, but increased sharply to 72% in those aged >45 days). This pattern may reflect cumulative exposure to the pathogen in the rearing environment or increased host susceptibility due to factors such as immunosuppression or management related stress with advancing age. This age-dependent distribution indicates that adult flocks, especially breeding and laying hens, serve as key populations for the transmission and persistence of *M. synoviae*.

The present survey identified a remarkably high co-infection rate of *C. baileyi* and *M. synoviae*. Among *C. baileyi* positive samples, 59.30% were concurrently positive for *M. synoviae*, a proportion that significantly exceeds random expectation, strongly suggesting a biological association between the two pathogens. These findings indicate that *C. baileyi* infection may play a critical role in promoting the pathogenesis of *M. synoviae*. This association likely arises from their shared infection routes, particularly via the respiratory tract, and more importantly, from the immunosuppressive properties of *C. baileyi* (Rhee et al., 1997; Rhee et al., 1998a, 1998b, 1998c; Hao et al., 2008). Previous studies have demonstrated that *C. baileyi* infection significantly suppresses the immune response to chickens to Newcastle disease vaccine (NDV) and increases susceptibility to NDV. The underlying mechanism may involve structural damage to the bursa of Fabricius and impaired proliferation and differentiation of B lymphocytes, thereby compromising humoral immunity (Rhee et al., 1998c; Eladl et al., 2014). Our study further suggests that the immunosuppressive microenvironment induced by *C. baileyi* facilitates the colonization of *M. synoviae* following infection.

Under clinical conditions, *M. synoviae* usually causes subclinical infections (Zhang et al., 2022; Awad et al., 2023). However, when co-infected with other respiratory pathogens such as IBV, the pathogenicity of *M. synoviae* in the host is markedly exacerbated (Kamathewatta et al., 2024; Manders et al., 2025). Such co-infections increase slaughter condemnation rates, reduced weight gain and feed conversion efficiency, and consequently result in considerable economic losses to the poultry industry. Based on the high co-infection rate of *M. synoviae* and *C. baileyi* observed in this study, we further investigated whether these two pathogens exert synergistic pathogenicity using an experimental model in which 3-day-old chicks were first infected with *C. baileyi* and then challenged with *M. synoviae* strains of different virulence. Among them, MSH is an attenuated strain derived from the virulent *M. synoviae* strain 86,079/7NS via chemical mutagenesis using N-methyl-N′-nitro-N-nitrosoguanidine (NTG), and is widely used for the control of *M. synoviae* in commercial poultry farms (Morrow et al., 1998; Klose et al., 2023). During co-infection with *M. synoviae* and *C. baileyi*, although the prepatent period of *C. baileyi* oocysts shedding was unchanged, the duration of oocyst shedding was significantly prolonged. In the single infection group, oocyst shedding was almost undetectable by 15 dpi, whereas in the co-infection group, shedding persisted until 17 dpi. This 2-day extension in shedding duration has important epidemiological implications under intensive farming conditions, as it greatly increases the opportunity for horizontal transmission of *C. baileyi* within flocks and may lead to more persistent and widespread outbreaks. This altered shedding pattern suggests that *M. synoviae* infection may interfere with the local or systemic immune response required for the host to clear *Cryptosporidium*, such as by impairing the integrity of immunity or the function of effector cells (Zhong et al., 2024). Furthermore, we compared the pathological lesions in the laryngeal and tracheal tissues induced by co-infection with *C. baileyi* and *M. synoviae*. Histopathological examination showed that single infection with *C. baileyi* only induced mild sloughing of laryngeal glandular epithelial cells and inflammatory cell infiltration. In contrast, co-infection with both pathogens resulted in qualitatively more severe lesions, including multifocal hemorrhages, marked focal inflammatory cell infiltration in the interacinar stroma, disorganization of the tracheal mucosal epithelium, and vascular congestion and dilation. This synergistic pathological effect is consistent with observations from co-infections of *M. synoviae* with other pathogens (Landman and Feberwee, 2004). Our study demonstrates that co-infection with *C. baileyi* and *M. synoviae* exacerbates tissue damage caused by *C. baileyi*.

Co-infection experiments also revealed that combined infection with *M. synoviae* and *C. baileyi* significantly increased the burden of *M. synoviae* in the host. At 7, 14, and 21 dpi, *M. synoviae* loads in choanal cleft swabs from the co-infection groups were significantly higher than those in the corresponding virulent strain single-infection groups. A higher pathogen load indicates enhanced tissue invasiveness, more intense release of inflammatory mediators, and ultimately more severe tissue injury. The more pronounced lesions observed in the air sacs, footpads, and joints of the co-infected chickens are likely attributable to the excessive proliferation of *M. synoviae* in these tissues. The synergistic pathogenic effects observed in the coinfection with *M. synoviae* and *C. baileyi* are somewhat comparable to those reported in other *M. synoviae* coinfection models. For example, coinfection with IBV and *M. synoviae* has been demonstrated to exacerbate *M. synoviae*-induced airsacculitis and arthritis (Landman and Feberwee, 2004), which aligns with our observation that *C. baileyi* aggravated lesions in the air sacs and footpads. Likewise, ILTV has been reported to enhance the pathogenicity of *M. synoviae* in commercial chicken flocks (Zorman et al., 2021). Collectively, these findings indicate that *M. synoviae* readily establishes synergistic interactions with a range of respiratory pathogens. Nevertheless, *C. baileyi* displays several unique characteristics in its synergistic pathogenesis that clearly distinguish it from viral coinfections. First, the parasitism of *C. baileyi* in the respiratory tract and bursa of Fabricius causes epithelial damage and exfoliation (Yang et al., 2016), which compromises the host’s primary physical barrier against *M. synoviae* colonization, thereby providing additional receptors and invasion routes. Furthermore, *C. baileyi* infection induces bursal atrophy and dysfunction, which impairs B-cell development and antibody production, thus weakening humoral immunity (Eladl et al., 2014). As an extracellular pathogen that mainly colonizes mucosal surfaces, *M. synoviae* relies heavily on effective mucosal antibody responses (e.g., sIgA) for clearance (Li et al., 2019; Xu et al., 2024; Wang et al., 2024). Suppression of humoral immunity allows *M. synoviae* to proliferate rapidly. Meanwhile, *C. baileyi* may also affect the function of immune cells such as macrophages and T cells, disturb the balance of cellular immunity, and create an immunosuppressive microenvironment favorable for the persistence of *M. synoviae*. Previous studies have reported that *C. baileyi* can enhance the replication of avian reovirus (Guy et al., 1988), which supports the hypothesis proposed in this study and indicates that its immunosuppressive effects are broad-spectrum. Finally, viral coinfections typically induce acute inflammatory responses, whereas *C. baileyi* acts through a dual mechanism: physical barrier disruption (respiratory epithelial injury) and immunomodulation (bursal dysfunction). This dual mechanism may explain why *C. baileyi* not only promotes the colonization of *M. synoviae* but also prolongs its own oocyst shedding period, forming a bidirectional synergistic cycle.

The attenuated MSH vaccine is commonly used for the control and prevention of *M. synoviae* in commercial poultry production (Noormohammadi et al., 2007; Omotainse et al., 2023). However, our data indicate that the immune protective efficacy of the MSH vaccine strain is significantly compromised under conditions of *C. baileyi* co-infection. The co-infection group not only exhibited higher *M. synoviae* loads but also developed footpad swelling and exacerbated airsacculitis, which are not typically observed in vaccinated chickens. This suggests that the immunosuppression induced by *C. baileyi* is sufficient to overcome the immune protection conferred by the vaccine. These findings pose a serious challenge to the current *M. synoviae* control strategy based on the MSH attenuated live vaccine. In flocks where *C. baileyi* is endemic or potentially present, chickens may remain inadequately protected despite standard *M. synoviae* vaccination and become more susceptible to subsequent infections due to immunosuppression. This may partially explain why *M. synoviae* related diseases still occur in vaccinated flocks under field conditions. Therefore, our study strongly supports a shift from a single-pathogen control strategy to an integrated management approach that considers pathogen interactions. Incorporating surveillance and control of *C. baileyi* into comprehensive health management programs, such as improving environment hygiene to reduce oocyst contamination and regularly monitoring parasitic infection status, may be crucial for enhancing the immune efficacy of *M. synoviae* vaccines and achieving stable disease control.

## 5 Conclusion

Through a large-scale epidemiological investigation, this study systematically revealed, for the first time, the high co-infection rate of *M. synoviae* and *C. baileyi* in poultry farms in Guangdong province. Using animal infection model, we further demonstrated that co-infection significantly promotes the *in vivo* proliferation, prolonged shedding, and exacerbated histopathological damage of both pathogens. These findings not only fill a knowledge gap in the field of co-infection research but also provide new insights into the interactive mechanisms of multiple pathogens in poultry. The results have important significance for guiding the accurate diagnosis of mixed infections in clinical practice, optimizing vaccination programs, and developing integrated prevention and control strategies, thus providing key scientific support for addressing the complex disease challenges currently encountered in poultry production.

## Data Availability

The original contributions presented in the study are included in the article/supplementary material, further inquiries can be directed to the corresponding authors.
